# Adiponectin Reduces Bone Stiffness: Verified in a Three-Dimensional Artificial Human Bone Model *In Vitro*

**DOI:** 10.3389/fendo.2018.00236

**Published:** 2018-05-14

**Authors:** Sigrid Haugen, Jianying He, Alamelu Sundaresan, Astrid Kamilla Stunes, Kristin Matre Aasarød, Hanna Tiainen, Unni Syversen, Bjørn Skallerud, Janne Elin Reseland

**Affiliations:** ^1^Department of Biomaterials, Institute for Clinical Dentistry, University of Oslo, Oslo, Norway; ^2^Department of Structural Engineering, Norwegian University of Science and Technology (NTNU), Trondheim, Norway; ^3^Department of Biology, Texas Southern University, Houston, TX, United States; ^4^Department of Cancer Research and Molecular Medicine, Norwegian University of Science and Technology (NTNU), Trondheim, Norway; ^5^St. Olav’s University Hospital, Trondheim, Norway; ^6^Department of Endocrinology, St. Olav’s University Hospital, Trondheim, Norway

**Keywords:** adiponectin, bone stiffness, bone remodeling, osteospheres, three-dimensional culturing system

## Abstract

Primary human osteoblasts and osteoclasts incubated in a rotating coculture system without any scaffolding material, form bone-like tissue that may be used to evaluate effects of various compounds on mechanical strength. Circulating adiponectin has been found to be negatively associated with BMD and strength and was therefore assessed in this system. Osteospheres of human osteoblasts and osteoclasts were generated with and without adiponectin. The osteospheres were scanned using micro-computed tomography, the mechanical properties were tested by flat punch compression using nanoindentation equipment, and the cellular morphology characterized by microscopy. The association between autologously produced adiponectin and biomechanical properties was further evaluated by quantitation of adiponectin levels using quantitative polymerase chain reaction (qPCR) and immunoassays, and identification of stiffness by bending test of rat femurs. The molecular mechanisms were examined *in vitro* using human bone cells. Mechanical testing revealed that adiponectin induced a more compliant osteosphere compared with control. The osteospheres had a round, lobulated appearance with morphologically different areas; inner regions containing few cells embedded in a bone-like material surrounded by an external area with a higher cell quantity. The expression of adiponectin was found to correlate positively to ultimate bending moment and ultimate energy absorption and deflection, on the other hand, it correlated negatively to bending stiffness, indicating autocrine and/or paracrine effects of adiponectin in bone. Adiponectin enhanced proliferation and expression of collagen, leptin, and tumor necrosis factor-alpha in osteoblasts and stimulated proliferation, but not the functional activity of osteoclasts. Our results indicate that both administration of adiponectin during osteosphere production and *in situ* elevated levels of adiponectin in rat femurs, reduced stiffness of the bone tissues. An increase in undifferentiated cells and extracellular matrix proteins, such as collagen, may explain the reduced bone stiffness seen in the osteospheres treated with adiponectin.

## Introduction

Cultivation of cells has traditionally been a cost-effective way to evaluate the results of a treatment without having to involve animals in larger and more expensive studies. Recently, there has been increasing evidence that three-dimensional (3D) culturing systems provide more accurate information than two-dimensional (2D) systems because of the more relevant architectural microenvironment that improves cell–cell interaction and cell-extracellular matrix (ECM) interaction (1–3). In bone, osteoblasts, osteocytes, and osteoclasts are the main cell types involved in building, maintenance and repair of bone, cooperating in a complex manner. During remodeling of bone, cells are situated in discrete anatomical multicellular units called the Basic Multicellular Unit (BMU), and 3D culturing systems have been created to mimic the BMU environment (4–9). Different scaffolding materials (natural and/or synthetic) have been used in the production and investigation of bone *in vitro* (4, 6, 10, 11). Recently, a model of bone formation has been presented, where two calcium phosphate scaffolds were used as anchors and cells grown on a fibrin gel cast in between (12). However, the presence of scaffolds makes it challenging to investigate the effect of a substance on the mechanical properties of the artificial bone cell constructs. Primary human osteoblast and osteoclast precursor cells can be mixed in a rotating cell culture system (RCCS) to generate 3D mineralized tissue constructs called osteospheres, where the cells form their own ECM components (9). In these osteospheres, osteoblasts and osteoclasts surround a porous mineralized matrix containing embedded osteocytes (9). These bone-like tissues may be used to test the effect of different substances on bone mechanical properties. Adiponectin has been shown to influence the metabolism and mechanical properties of bone (13–15) and was therefore investigated in this study.

Adiponectin was identified in 1995 by several research groups (16–19). It occurs in different forms in plasma including a globular domain and full-length adiponectin that may aggregate into oligomer forms including a trimer, a hexamer, and heavier, multimer forms. The functions of adiponectin vary dependent on the target organ and its isoforms (20).

The main physiological role of adiponectin is considered to be related to metabolic regulation and energy homeostasis (20, 21). Adiponectin levels are abundant in circulation relative to other hormones, and although it is produced by adipose tissue, plasma concentrations of adiponectin has been negatively associated with increasing obesity (22) and type 2 diabetes (23, 24). Furthermore, serum adiponectin has been shown to be a predictor of fractures in type 2 diabetic patients (25).

An inverse correlation between circulating adiponectin levels and different bone parameters, including BMD (14, 15), axial stiffness and maximal load (26), has been reported in various patient groups in humans. However, the effects of adiponectin on bone in animal studies are inconclusive (13). Shinoda et al. reported no particular changes in the bone phenotype of adiponectin-knockout (APN-KO) mice (27), while Kajimura et al. observed stiffer bones in the same type of animals (APN-KO) (28). To further complicate the matter, exercise has been found to increase both adiponectin levels and bone mass in rats (29).

Adiponectin may act *via* its receptors, adiponectin receptor 1 and adiponectin receptor 2, that have been found to be expressed in osteoblasts (30) and osteoclasts (31). However, studies have demonstrated that adiponectin can affect osteoblasts through both direct and indirect mechanisms including; favoring of osteogenesis in mesenchymal progenitor cells (32, 33), stimulation of osteoblast proliferation and differentiation (34–37), and decrease in the sympathetic tone in osteoblasts *via* neurons of the locus coeruleus resulting in increased bone mass (28, 33). The globular domain of adiponectin shows structural similarity to tumor necrosis factor-α (TNF-α), receptor activator of nuclear factor κB ligand (RANKL), and osteoprotegerin (OPG), which all are involved in the regulation of osteoclastogenesis (38). The described effects of adiponectin on osteoclastogenesis are multiple and deviating. Most studies report suppressive (33, 36, 39–41), while others enhancing effects (42, 43) of adiponectin on osteoclast differentiation. Based on the previous observations our hypothesis was that osteospheres produced in a rotational coculture *in vitro* could be used as a model system to study changes in bone mechanical properties. Since circulating adiponectin levels are found to be negatively associated with fracture risk, we wanted to investigate both the effects of adiponectin administration *in vitro*, and changes in the level of autologously produced adiponectin *in vivo*, on the biomechanical properties of bone. Moreover, we aimed to identify the molecular mechanisms of adiponectin on single cell cultures of human osteoblasts and osteoclasts.

## Materials and Methods

### Preparation of Osteospheres From Primary Human Osteoblasts and Osteoclasts

Commercially available primary human osteoblasts (NHO) (Cambrex Bio Science, Walkersville, MD, USA) were cultured in osteoblast growth medium (OGM) supplemented with 10% fetal calf serum (FCS), 5 µM sodium ascorbate and 100 µg/mL penicillin/100 IU μg/mL streptomycin (Lonza, Allendale, NJ, USA). NHOs were mixed in a 4:1 ratio with normal osteoclast precursor cells derived from human peripheral blood mononuclear cells (PBMC) (Lonza) cultured in osteoclast precursor basal medium containing 10% FCS, human recombinant macrophage colony-stimulating factor (M-CSF) (50 ng/mL), and human recombinant RANKL (50 ng/mL) (Lonza), with and without adiponectin (0.08 µg/mL) (BioVision Inc., Milpitas, CA, USA). At day 7, media were supplemented with hydrocortisone hemisuccinate (200 nM) and β-glycerophosphate (10 mM) (Lonza). Osteospheres were generated using disposable 10 mL high-aspect-ratio vessels (Synthecon, Houston, TX, USA) in an RCCS operated in a 5% CO_2_ humidified tissue culture incubator maintained at 37°C (9). Osteospheres were harvested after 21 days in the RCCS. The adiponectin concentration was based on Berner et al. (30), where adiponectin concentration in the mice bone marrow was found to be between 0.1 and 0.15 µg/mL. Osteospheres of various sizes were generated and only the largest spheres (~0.5–1 mm) were used for mechanical testing. The osteospheres used for histology were harvested and stored in −70°C until further use.

### Micro-Computed Tomography of Osteospheres

The osteospheres from primary human osteoblasts and osteoclasts were scanned using a SkyScan 1176 (Bruker, Kontich, Belgium) at 9 µm voxel size. 3D reconstructions were made using Medical Image Segmentation for Engineering on Anatomy (MIMICS) software (Materialize HQ, Leuven, Belgium). The reconstructed geometries were used to identify global measures, such as overall height and extension and shape of the cross section at the equatorial plane. These data were employed in finding stress and strain measures from the measured global force and displacement in the mechanical testing of the osteospheres (see next section).

### Mechanical Testing of Osteospheres

**Figure 1:**
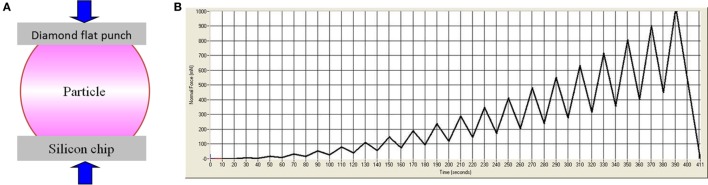
A schematic illustration of the flat punch method for compression test of particle-like materials is shown in panel **(A)** and the loading scheme is illustrated in panel **(B)**.

The mechanical response of the osteospheres at room temperature was characterized by nanoindentation using Hysitron TI950 TriboIndenter^®^ (Hysitron, Minneapolis, MN, USA). Because of irregular geometry of samples, the conventional nanoindentation was not applicable. Instead, a so-called flat punch method for compression test of particle-like materials was used (44). The osteospheres were placed on a silicon chip and compressed with a diamond flat punch with a diameter of 1.08 mm, comparable with sample size, see Figure 1A for an illustration of the setup and boundary conditions. The maximum load 1 N was applied to each sample. The predefined loading function consisted of 20 partially loading–unloading cycles; a linear loading segment to peak load in 10 s, a 0.1 s holding segment at peak load, and a 10 s linear unloading segment within one cycle, see Figure 1B for the loading scheme (including the loading rate). The cyclic loading condition was used to counterbalance the viscoelastic effect of bone materials. The real-time displacement of the samples was recorded and analyzed. To remove some of the geometry effects on the mechanical response of the spheres, a simple conversion to nominal stress and strain was carried out. Based on the reconstructed MIMICS images, the applied force was divided by elliptical cross section area at equatorial plane to assess stress (i.e., stress = punch force/π*ab*, where *a* and *b* are the magnitude of the semi-axes), and the resultant displacement was divided by height of specimen to get a strain measure (i.e., strain = global displacement/height of sphere). The largest spheres (~0.5–1 mm) were chosen to obtain easier handling during experimental preparation. Due to large mass, the mechanical parameters obtained from these were based on average properties of more material, and the data less influenced by local artifacts.

### Microscopy Analysis of Osteospheres

Osteospheres were removed from RCCS for histologic characterization after 21 days of culturing, fixed in 4% formaldehyde (VWR, Radnor, PA, USA), dehydrated in ascending concentrations of ethanol and embedded in paraffin for histological analysis. Sections were cut and mounted on glass slides. Before staining, sections were de-paraffinized and rehydrated through graded ethanol rinses. Slides were stained with either Hematoxylin and Eosin (i.e., cell nuclei) or Alizarin Red (i.e., calcium) stain using standard protocols and imaged using a Leica DM RBE microscope (Leica, Wetzlar, Germany) with a digital camera. Original images were taken with resolution of 1.08 pixels per micron.

De-paraffinized and rehydrated sections were stained to detect cell nuclei and the protein periostin by confocal microscopy. Antigen retrieval was carried out with 10 mM citrate buffer pH 6.0 with 0.05% Tween20, 30 min at 95°C, before cooling to room temperature and permeabilizing with 0.3% Triton X-100 for 30 min. After washing with PBS, sections were blocked in 10% normal goat serum (NGS) (Abcam, Cambridge, UK) with 0.1% Triton X-100 (Sigma-Aldrich, St. Louis, MO, USA) in PBS for 1 h room temperature. Sections were immunolabeled with rabbit anti-periostin (ab14041, Abcam) diluted 1:300 in 2% NGS in PBS at 4°C overnight, washed three times with PBS, then incubated with Alexa488 goat anti-rabbit secondary antibody (ThermoFisher, Waltham, MA, USA) diluted 1:400 in 4% NGS in PBS, 1 h, room temperature. After washing three times with PBS, sections were co-stained with 300 nM 4′,6-diamidino-2-phenylindole, dihydrochloride (DAPI) (Sigma-Aldrich) for 30 min and mounted with Prolong Diamond (ThermoFisher, Waltham, MA, USA). Slides were viewed with a 10×/0.40 HCX PL APO CS objective lens on a Leica SP8 confocal microscope (Leica) using 405 and 488 nm excitation, and 410–470 and 500–550 nm emission filters for DAPI and Alexa488, respectively. Original images were taken with resolution of 2.77 pixels per micron. Images were viewed and processed in ImageJ (45).

### Relationship Between Adiponectin Expression and Biomechanical Data of Rat Femurs

Data on rat bones were retrieved from three studies on female Fisher 344 rats given the following PPAR agonists: Wyeth 14643 (90.0 mg/kg/day Wyeth 14643) (ChemSyn Laboratories, USA) (unpublished data), fenofibrate and pioglitazone (46), and tetradecylthioacetic acid (TTA) (47) dissolved in methylcellulose, or methylcellulose alone, for 4 months as described previously (46, 47). All animal experiments were approved by the Regional Animal Research Authority at the Norwegian University of Science and Technology, and the Federation of Laboratory Animal Science Associations guidelines were followed. Blood was collected by heart puncture during the final anesthesia, and plasma samples were stored at −80°C until analyzes. Femurs from 23 animals were randomly picked for biomechanical analysis from the different treatment groups in three larger animal studies (fenofibrate *n* = 8; Wyeth *n* = 6; TTA *n* = 3; pioglitazone *n* = 3; untreated *n* = 3). The femurs were fractured 18.7 mm from the femoral condyles in the so-called three point cantilever bending setup that has been described in detail elsewhere (48, 49). Briefly, the proximal femur was fixed in a clamp, and bending of the distal part of the femur was performed by a rotating wheel with a fixed cam applying bending force to the femoral condyle with a fulcrum positioned 18.7 mm anteriorly from the condyle acting as the third point of force application. All tests were done at a loading rate of 0.095 rad/s (5.43°/s), and the load was applied until fracture at the fulcrum occurred. The load in the test apparatus, a MTS 858 Mini Bionix^®^ Axial/Torsional Test System (MTS Systems Corporation, MN, USA), was measured with an MTS Test Star TM Sensor Cartridge Force 250 N load cell and registered in MTS Test Star II software. Ultimate bending moment (*M*) was calculated as the ultimate load before failure multiplied by the moment arm by which the load was applied (Nm). Ultimate energy absorption, stiffness, and deflection were read directly or calculated from computer recordings (48). After mechanical testing, the femurs were homogenized in liquid N_2_, and 200 mg tissue of each sample was aliquoted in Trizol (Invitrogen, Carlsbad, CA, USA). Messenger ribonucleic acid (mRNA) and proteins were extracted according to the manufacturers’ instructions (Invitrogen). The concentration of adiponectin in plasma and in protein extracts from femurs was quantified by a Mouse Adiponectin Radioimmunoassay (RIA) kit (Linco, St. Charles, MI, USA) and a Mouse adiponectin enzyme-linked immunosorbent assay (ELISA) kit (Linco, St. Charles, MI, USA), respectively. Relative abundance of adiponectin was calculated as ratios of the total protein content [25 μL of each sample diluted 1:10 using Bicinchoninic acid (BCA) Protein Assay Kit (Pierce Biotechnology, Rockford, IL, USA)].

### Identification of the Effect of Adiponectin on Human Bone Cells

NHOs from both femur and tibia of different donors (Cambrex Bio Science) were grown in OGM (Cambrex Bio Science). The cells were incubated with either 0.08, 0.4, or 2 µg/mL recombinant adiponectin (R&D Systems, Minneapolis, MN, USA) for 1 and 3 days, and the effect on incorporated ^3^H-thymidine was calculated relative to untreated cells at the same time points. Effect on proliferation rate of NHO cells was measured after a 12 h pulse with ^3^H-thymidine as previously described (50).

The effect of adiponectin (0.08, 0.4, and 2 µg/mL) on differentiation of osteoblasts was measured after 1, 3, 7, and 14 days of incubation. The concentration of various bone markers secreted in the cell culture medium was evaluated, and the phenotype of the cells was characterized based on the mRNA expression levels of runt-related transcription factor 2 (*RUNX2*), collagen type I alpha 1 (*COLIA1*), osteocalcin (*OC*) (*BGLAP*), and CD44 (*CD44*).

The effect of adiponectin (0.08, 0.4, and 2 µg/mL) on calcium (Ca^2+^) deposition in primary human osteoblasts was measured after 21 days of incubation and compared with unexposed controls. The cells were washed with PBS, fixed in 95% ethanol and stained with 1% Alizarin Red (Sigma-Aldrich, St. Louis, MO, USA) as described elsewhere (51). Alizarin Red was extracted with cetylpyridinium chloride (Sigma-Aldrich) at room temperature, measured spectrophotometrically at 562 nm (ELx800 Absorbance Reader, BioTek instruments, Winooski, VT, USA) and presented relative to untreated cells.

Osteoclasts were differentiated from PBMC isolated from buffy coat, performed essentially as described by Bøyum (52). Cells were seeded into 24-well dishes, 5.0 × 10^5^ cells/well in MEMα/10% FCS including M-CSF and RANKL (50 ng/mL of both), and dexamethasone (0.1 µM) (Sigma-Aldrich) with or without 0.1 µg/mL adiponectin (Phoenix Pharmaceuticals, Belmont, CA, USA). Assays were performed in triplicate. The medium was replaced at days 6 and 9. After 12 days, the cells were stained for tartrate resistant acid phosphatase (TRAP) using an acid phosphatase kit (Sigma-Aldrich), as described by the manufacturer. TRAP-positive, multinuclear (three or more nuclei) cells were regarded as genuine osteoclasts. To investigate direct osteoclast activity, a pit resorption assay was performed. Osteoclast activity (resorption) was evaluated by analyzing the amount of digested area by osteoclasts seeded and differentiated on hydroxyapatite disks (BD Biosciences, ON, Canada). PBMC were seeded on hydroxyapatite disks in 24-well dishes in osteoclast differentiation media with or without adiponectin, as described earlier. After 12 days, the amount of digested area was measured using a Bioquant scanner (Bioquant Image Analysis, Nashville, TN, USA).

### Quantification of Secreted Proteins in Cell Culture Media

Before analyses, aliquots of the cell culture medium were concentrated 10-fold using centrifugation filters with 3 kDa cutoff (VWR international, Radnor, PA, USA) according to manufacturer’s instruction. Multi-analyte profiling of the protein level in concentrated cell culture medium of primary human osteoblasts was performed on Luminex 200 system (Luminex, Austin, TX, USA) using xMAP technology. Acquired fluorescence data were analyzed by the xPONENT 3.1 software (Luminex). The effect of adiponectin on the secretion of bone markers to the culture medium was measured using the Milliplex Human Bone Panel kit [interleukin-1 (IL-1β), interleukin-6 (IL-6), OPG, OC, leptin, osteopontin (OPN), parathyroid hormone, TNF-α, adrenocorticotropic hormone, Dickkopf-related protein 1 (DKK1), sclerostin (SOST), and insulin] (Millipore, Billerica, MA, USA) after 1, 3, 7, and 14 days of culture. All analyses were performed according to manufacturers’ protocols.

### Isolation of mRNA From Cells

Cells were lysed in lysis/binding buffer [100 mM Tris–HCl, pH 8.0, 500 mM LiCl, 10 mM ethylenediaminetetraacetic acid, pH 8.0, 0.5 mM dithiothreitol, and 1% sodium dodecyl sulfate (Sigma-Aldrich)]. mRNA was isolated using magnetic beads [oligo (dT) 25] as described by the manufacturer (Dynal AS, Oslo, Norway). Beads containing mRNA were suspended in 10 mM Tris–HCl, pH 8.0, and stored at −70°C until use. 10 µL of the mRNA-containing solution was applied directly to obtain a first-strand complementary cDNA using the iScript cDNA Synthesis Kit containing both oligo (dT) and random hexamer primers (Bio-Rad, Hercules, CA, USA).

### Quantitative Polymerase Chain Reaction (qPCR) Quantification of Gene Expression

Gene expression was monitored using iCycler iQ and CFX connect (Bio-Rad). The 2× iQ SYBR Green Supermix was based on iTaq DNA polymerase (Bio-Rad). cDNA samples (1 µL for a total volume of 25 µL per reaction) were analyzed both for the genes of interest and the reference genes [β-actin and glyceraldehyde-3-phosphate dehydrogenase (*GAPDH*)]. The cycling profile was as follows: denaturing at 94°C for 5 min followed by 40 cycles of annealing at 57°C for 30 s, primer extension at 72°C for 30 s, and denaturing at 95°C for 30 s. Finally, one cycle for 3 min of extension completed the reaction. Reactions were replicated twice on a 96-well plate. Cycle threshold (Ct) values were obtained graphically. Gene expression was normalized to β-actin and *GAPDH* and presented as ΔCt values. Comparison of gene expression between control and treated samples was derived from subtraction of control ΔCt values from treatment ΔCt values to give a ΔΔCt value, and relative gene expression was calculated as 2^−ΔΔCt^. The efficiency of each set of primers was always higher than 90%. The sequences of the oligonucleotides are shown in Table 1.

**Table 1:** Description of the rat (r) and human (h) oligonucleotide primers used for qPCR.

Protein	Gene	Primer sequence (5′–3′)
Adiponectin	r-*ADIPOQ* r-*ADIPOQ*	f AATCCTGCCCAGTCATGAAG r CATCTCCTGGGTCACCCTTA

Glyceraldehyde-3-phosphate dehydrogenase	r-*GADPH* r-*GADPH*	f ATGATTCTACCCACGGCAAG r CTGGAAGATGGTGATGGGTT

β-Actin	r-*ACTB* r-*ACTB*	f CCTCTATGCCAACACAGT r AGCCACCAATCCACACAG

Collagen type I alpha 1	h-*COL1A1* h-*COL1A1*	f CCAAATCCGATGTTTCTGCT r CATCTCCCCTTCGTTTTTGA

Alkaline phosphatase	h-*ALPL* h-*ALPL*	f AGACTGCGCCTGGTAGTTGT r GACAAGAAGCCCTTCACTGC

Runt-related transcription factor 2	h-*RUNX2* h-*RUNX2*	f GCCTTCAAGGTGGTAGCCC r CGTTACCCGCCATGACAGTA

CD44	h-*CD44* h-*CD44*	f CAAGTTTTGGTGGCACACAGC r GAAGCAATATGTGTCATACTGGGAG

Glyceraldehyde-3-phosphate dehydrogenase	h-*GADPH* h-*GADPH*	f TGCACCACCAACTGCTTAGC r GGCATGGACTGTGGTCATGAG

β-Actin	h-*ACTB* h-*ACTB*	f CTGGAACGGTGAAGGTGACA r AAGGGACTTCCTGTAACAATGCA

Osteocalcin	h-*BGLAP* h-*BGLAP*	f CACTACCTCQCTQCCCTCC r QAAQCCCAQCQQTQCA

*qPCR, quantitative polymerase chain reaction; f, forward; r, reverse*.

### Statistics

The differences between the mechanical variables were tested with one way ANOVA. The relationship between mechanical data and adiponectin expression was tested by Spearman rank order correlation. Comparison between data obtained from the various groups and treatments by ELISA and RIA measurements was performed using the non-parametrical tests one way ANOVA or Mann–Whitney *U* test. qPCR (ΔΔCt values), Luminex, proliferation, and Alizarin Red quantification data were analyzed by *t*-test or Mann–Whitney *U* test. A probability of less than or equal to 0.05 was considered significant.

## Results

### Adiponectin Reduces the Stiffness of Osteospheres

**Figure 2:**
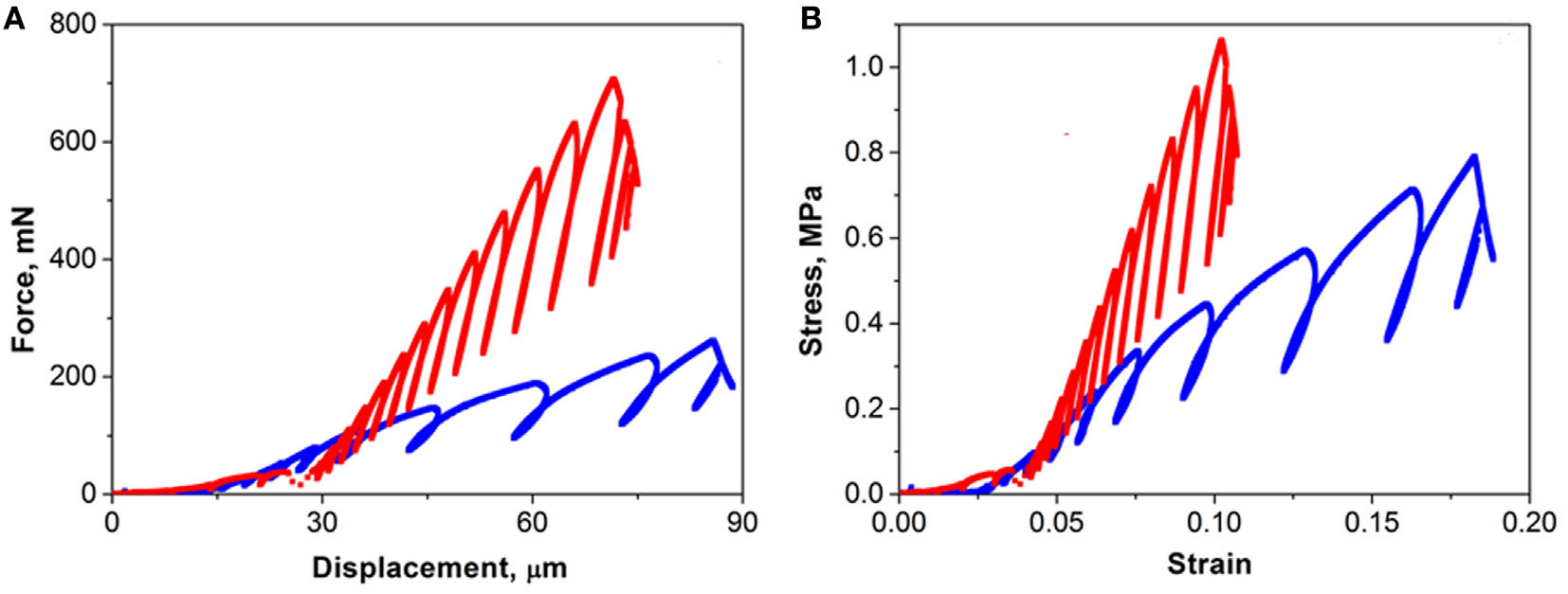
Compression force–displacement curves and stress–strain relationship of osteospheres treated with (blue line) and without (red line) adiponectin. The global response of the spheres, i.e., nanoindentation force versus displacement is shown in panel **(A)**. The corresponding nominal stress–strain response is illustrated in panel **(B)**. Notable difference in the stiffness of the two osteospheres was observed as the tangent stiffness of the adiponectin-treated osteospheres was considerably lower than that of the control sample.

Micro-computed tomography scans identified irregularly shaped osteospheres (0.5–1 mm). The global response of the spheres, i.e., the flat punch force versus displacement. The global response of the spheres, i.e., nanoindentation force versus displacement, is shown in Figure 2A whereas the corresponding nominal stress–strain response is shown in Figure 2B. The tangent stiffness was obtained by the line connecting the stress at each cyclic peak load and found to be 6.47 and 16.93 MPa for osteospheres with and without adiponectin exposure, respectively. Elastic stiffness of the osteospheres was estimated from the unloading sections of the stress–strain curves using the sixth unloading/loading cycle and was much lower for the adiponectin-treated osteosphere (17.4 MPa) compared with control (37.6 MPa). The pop-in on initial section of the loading curve of the control was considered to arise from collapse of the pore found within this osteosphere.

### Morphological Characteristics of Osteospheres

**Figure 3:**
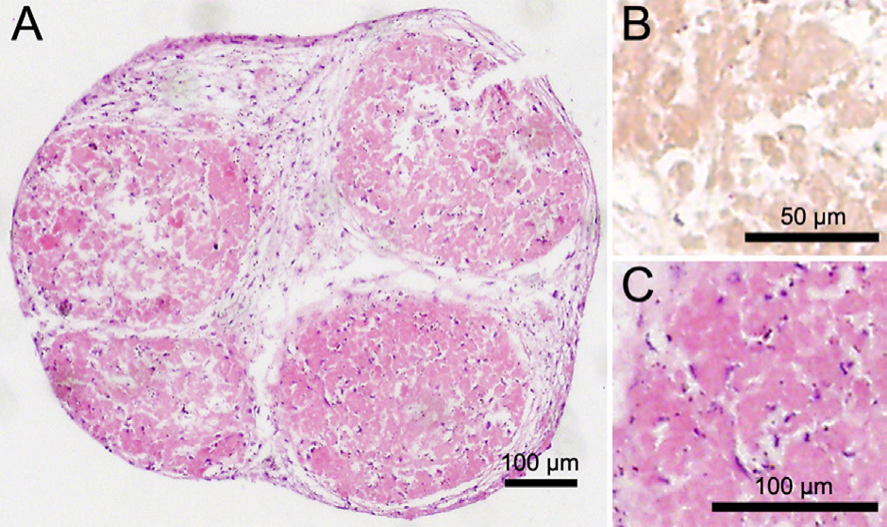
The osteospheres has a lobulated appearance with two morphologically different regions shown here in section stained with hematoxylin and eosin (scale bar = 100 µm) **(A)**. In this osteosphere, the outer region of the sphere and region between the lobuli the osteosphere contained cells **(A)**. Mineralization of the extracellular matrix is visualized by Alizarin Red staining **(B)**. In the inner region of the lobuli, sections show few cells embedded in bone-like material **(C)**.

**Figure 4:**
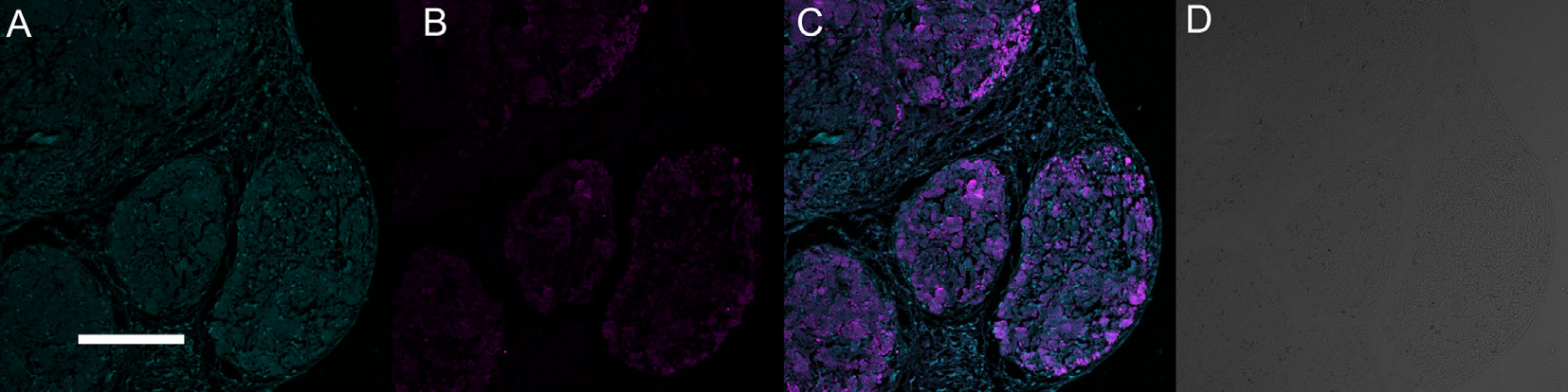
Immunohistochemical characterization of cell nuclei and periostin in paraffin embedded sections of 21-day-old mineralized osteospheres stained with 4′,6-diamidino-2-phenylindole, dihydrochloride (nuclei) (green) **(A)** and periostin (purple) **(B)** and both **(C)** (scale bar = 200 µm). The outer region contained cells whereas the inner region was made up of osteoblasts embedded in self-produced extracellular matrix with collagen indirectly visualized by periostin **(C)**. The last image **(D)** is a bright-field version.

Sections of 21-day-old mineralized constructs showed round osteospheres with a lobulated appearance. This may be explained by multiple smaller osteospheres that seemed to have merged (Figure 3A). It consisted of distinct units previously described by Clarke et al. as osteoclasts and osteoblasts that are situated in the outer region of the spheres (9). Alizarin Red staining showed an inner region of the spheres containing calcium (Figure 3B), with individual cells trapped within the tissue as indicated by hematoxylin (Figure 3C) and DAPI (Figures 4A,C) staining of cell nuclei. The osteoblasts in the inner region of the osteospheres produced collagen as expected, shown by periostin positive staining (Figures 4B,C).

### Relationship Between Adiponectin Expression and Mechanical Properties of Rat Femurs

The biomechanical data are given in Table 2. There were no significant differences in ultimate bending moment (*p* = 0.257), ultimate energy absorption (*p* = 0.273), bending stiffness (*p* = 0.692), or deflection (*p* = 0.071) of the femur between the different experimental groups tested (Table 2).

**Table 2:** Mechanical data for bending of rat femurs (*n* = 23).

	*M* (Nm)	Ultimate energy absorption (J)	Stiffness (Nm/°)	Deflection (°)
All groups (*n* = 23)	45.7 ± 3.7	1.07 ± 0.09	1.03 ± 0.10	16.2 ± 1.9
Control	48.4 ± 1.6	1.13 ± 0.04	0.98 ± 0.18	17.9 ± 3.0
TTA	43.3 ± 2.0	1.01 ± 0.05	1.07 ± 0.07	14.8 ± 1.0
Pioglitazone	43.4 ± 5.6	1.01 ± 0.13	1.08 ± 0.13	14.5 ± 1.3
Wyeth 14643	45.0 ± 2.9	1.05 ± 0.07	1.03 ± 0.10	15.9 ± 1.6
Fenofibrate	47.0 ± 3.9	1.10 ± 0.09	1.00 ± 0.07	17.0 ± 1.5

*Data presented as mean ± SD*.

*TTA, tetradecylthioacetic acid*.

**Figure 5:**
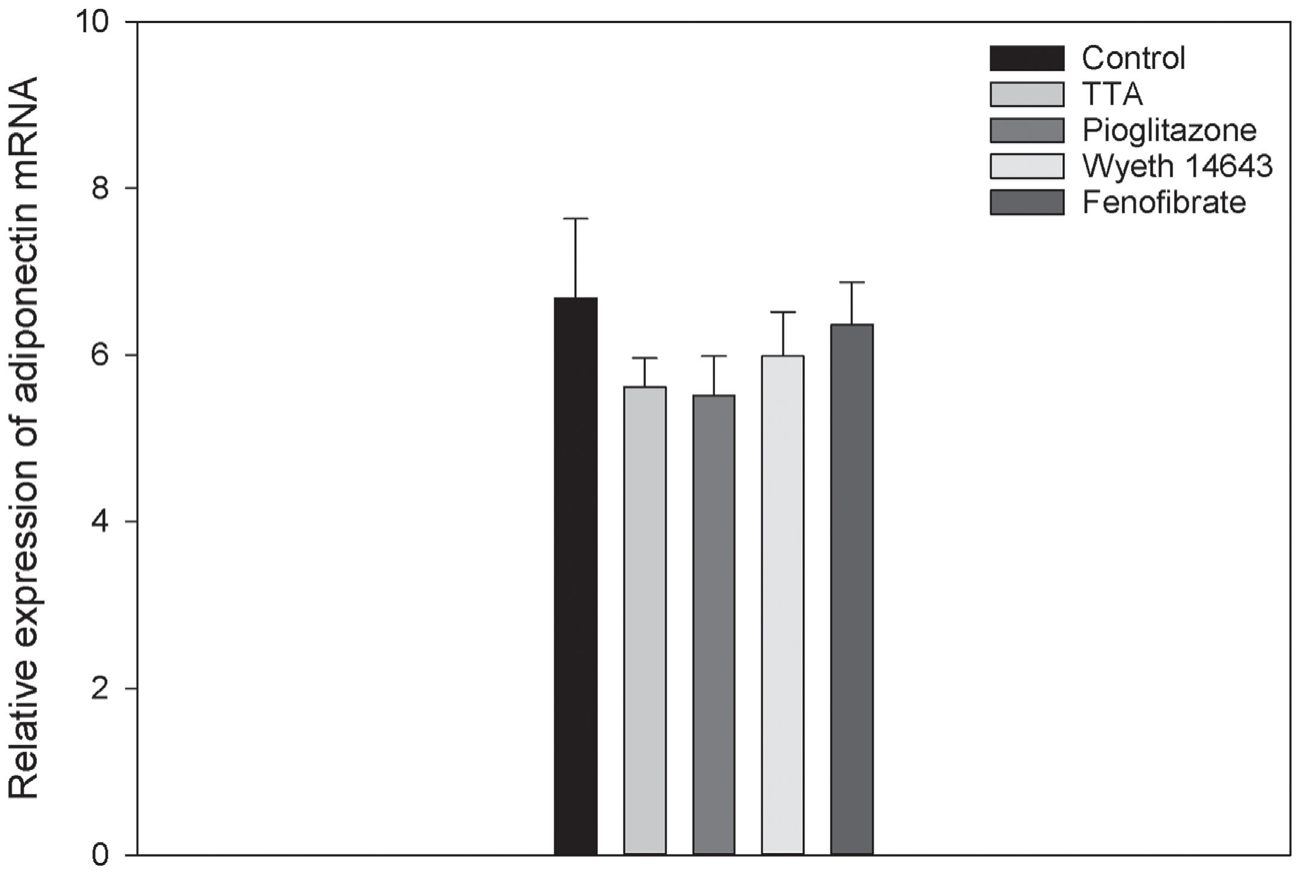
The relative expression of adiponectin messenger ribonucleic acid (mRNA) in rat femur in the different treatment groups are shown. There were no significant differences between the groups. Data are expressed as mean ± SD.

The relative expression of adiponectin mRNA in femur of the rats were measured and the difference between the groups was not significant (*p* = 0.066), possibly due to the limited number of samples in each group (Figure 5).

The correlation test between adiponectin mRNA expression levels and mechanical parameters show that the relative expression of adiponectin mRNA in the femur positively correlated to ultimate bending moment, ultimate energy absorption and deflection, and negatively to bending stiffness (Table 3).

**Table 3:** Correlation between adiponectin messenger ribonucleic acid (mRNA) and protein in bone, adiponectin in plasma, and mechanical properties in rats (*n* = 23).

		*M* (Nm)	Ultimate energy absorption (J)	Stiffness (Nm/°)	Deflection (°)
mRNA (bone)	*r*	0.773	0.733	**−**0.506	0.988
	*p*	**<**0.001	**<**0.001	0.014	**<**0.001
Adiponectin (bone)	*r*	0.126	0.126	−0.0845	0.0647
	*p*	0.561	0.561	0.696	0.764
Adiponectin (plasma)	*r*	−0.029	−0.252	−0.164	−0.164
	*p*	0.890	0.242	0.450	0.450

*Correlation coefficient: *r* (Spearman rank order correlation)*.

The mean concentration of adiponectin isolated from the bone samples was 38.2 ± 10.5 ng/200 mg bone mass (*n* = 23); 48.4 ± 8.0 ng in the control group, 38.3 ± 10.3 ng in the group receiving the PPARpan agonist TTA, 42.4 ± 9.3 ng in the groups receiving the PPAR gamma agonist pioglitazone, and 37.8 ± 7.1 ng and 31.4 ± 13.6 ng, respectively, in the groups receiving the PPAR alpha agonists fenofibrate and Wyeth. No significant differences were found between the groups.

The quantified adiponectin levels in the protein fractions isolated from femur were negatively associated with stiffness; however, not statistically significant (Table 2).

No significant relationship between circulating plasma adiponectin levels and the bone biomechanical data was found (Table 3).

### Adiponectin Enhanced Bone Cell Proliferation and the Expression of Collagen mRNA

**Figure 6:**
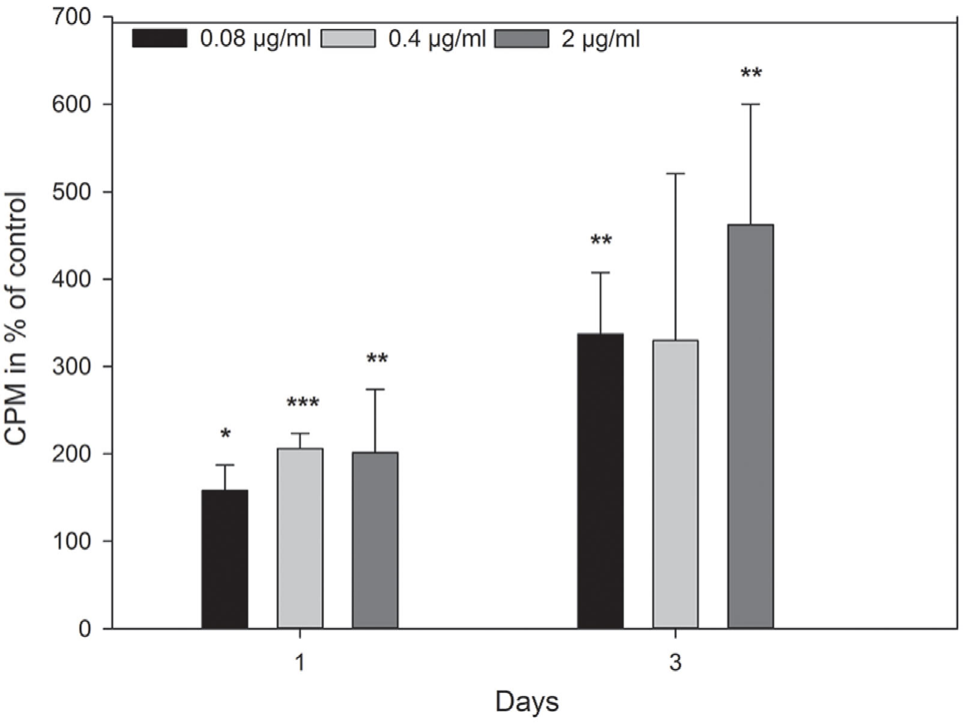
Effect of adiponectin on cell growth of primary human osteoblasts after 1 and 3 days measured as ^3^H-thymidine incorporation in counts per minute (CPM). Adiponectin (0.08, 0.4, and 2 µg/mL) significantly enhanced the proliferation of primary human osteoblasts on both time points compared with control. Values represent the mean ± SD of one experiment with three parallels. Significantly different from control at *p < 0.05, **p < 0.01, and ***p < 0.001.

The effect of adiponectin (0.08, 0.4, and 2 µg/mL) on the proliferation rate of osteoblasts was calculated as counts per minute in percent of untreated control at each time point (Figure 6). Enhanced proliferation was observed after both 1 and 3 days of incubation compared with control.

**Figure 7:**
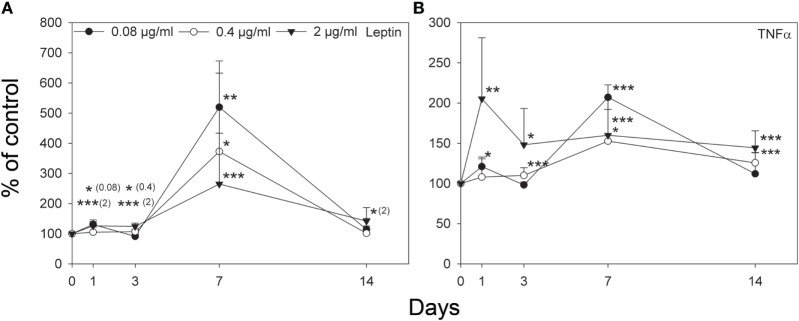
Secretion of leptin **(A)** and tumor necrosis factor-α (TNF-α) **(B)** to culture medium from primary human osteoblasts treated with and without adiponectin is presented in percentage of control at 1, 3, 7, and 14 days. Adiponectin (0.4 and 2 µg/mL) treated osteoblasts showed upregulated levels of leptin **(A)** and TNF-α **(B)**. Values represent the mean of two donors ± SD. Significantly different from control at *p < 0.05, **p < 0.01, and ***p < 0.001.

The levels of selected protein markers associated with differentiation and mineralization of NHOs were quantified, and OC, OPN, SOST, and DKK1 were not affected by adiponectin treatment, whereas leptin secretion was more than threefold enhanced (*p* < 0.01) on day 7 by the lowest dosage (0.08 µg/mL) and more than twofold (*p* < 0.001) by the highest dosage (2 µg/mL) of adiponectin (Figure 7A). Adiponectin (2 µg/mL) treatment resulted in an initial twofold increase in the release of TNF alpha (*p* < 0.01), whereas after 7 days of incubation all dosages of adiponectin (0.08, 0.4, and 2 µg/mL) significantly increased TNF alpha levels (*p* < 0.001, *p* < 0.05, and *p* < 0.001, respectively), and after 14 days of culture a significantly higher amount was detected compared with control with 0.4 and 2 µg/mL adiponectin (*p* < 0.001 and *p* < 0.001, respectively) (Figure 7B).

**Figure 8:**
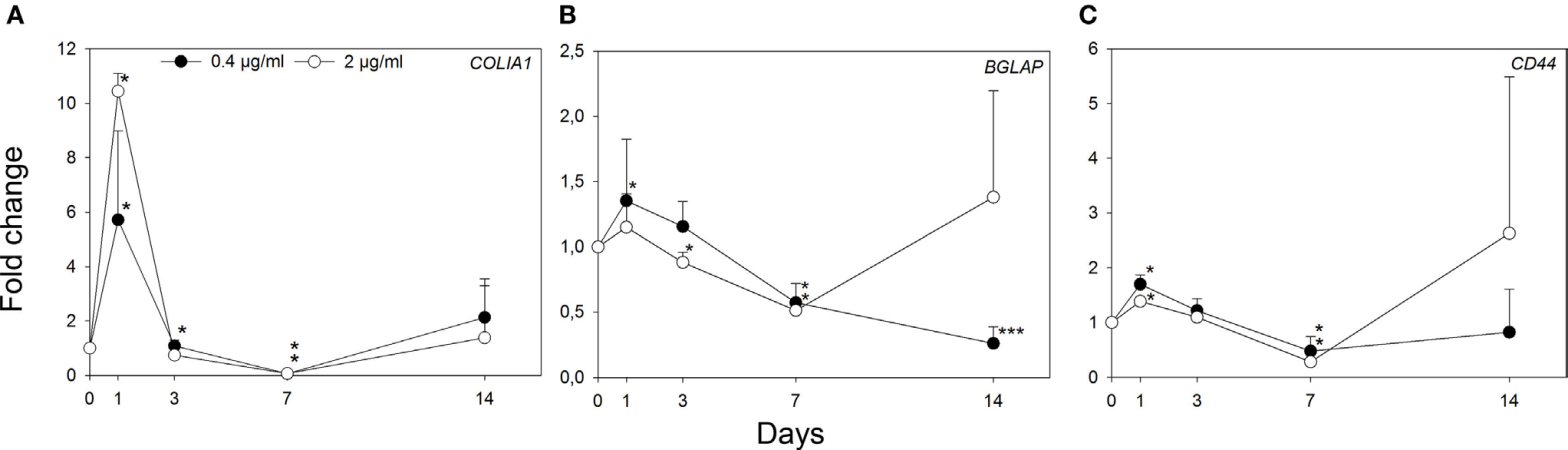
Relative messenger ribonucleic acid expression levels for *COLIA1* **(A)**, *BGLAP* **(B)**, and *CD44* **(C)** in human primary osteoblasts cultured with 0.4 and 2 µg/mL adiponectin normalized to reference genes *GAPDH* and *ACTB* and presented as fold change relative to controls. Adiponectin enhanced expression of *COLIA1* **(A)** more than eightfold compared with untreated cells after 1 day. Values represent the mean ± SD. Significantly different from control at *p < 0.05 and ***p < 0.001.

The mRNA expression levels of *COLIA1, BGLAP*, and *CD44* in NHOs is shown for two adiponectin concentrations (0.4 and 2 µg/mL) at selected time points (1, 3, 7, and 14 days) (Figure 8). Incubation with 2 µg/mL adiponectin induced a more than eightfold enhancement of *COLIA1* expression (*p* < 0.05) compared with unexposed cells on day 1 (Figure 8A).

**Figure 9:**
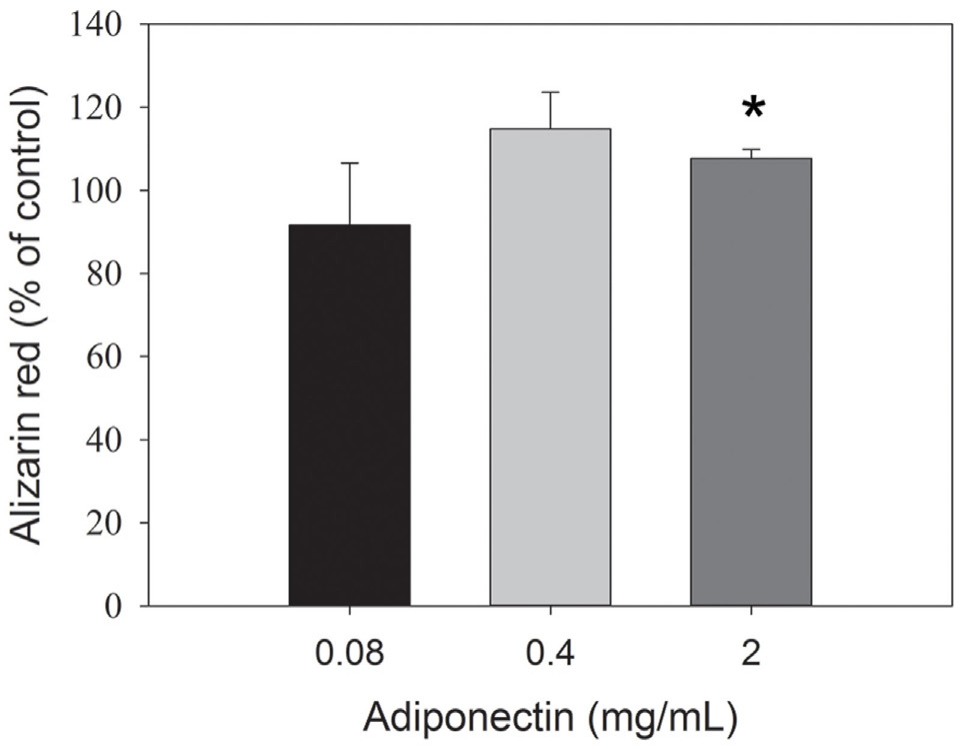
Quantification of Ca^2+^ deposits from primary human osteoblasts after 21 days of adiponectin treatment. Ca^2+^ deposition was significantly enhanced from osteoblasts by adiponectin (2 µg/mL) compared with untreated control cells. Data presented as means ± SD. Significantly different from untreated controls at *p < 0.05.

High dosage of adiponectin (2 µg/mL) enhanced the Ca^2+^ deposition in osteoblasts after 21 days of incubation, while no statistically significant difference was detected for the lower adiponectin concentrations in comparison to the untreated control (Figure 9).

**Figure 10:**
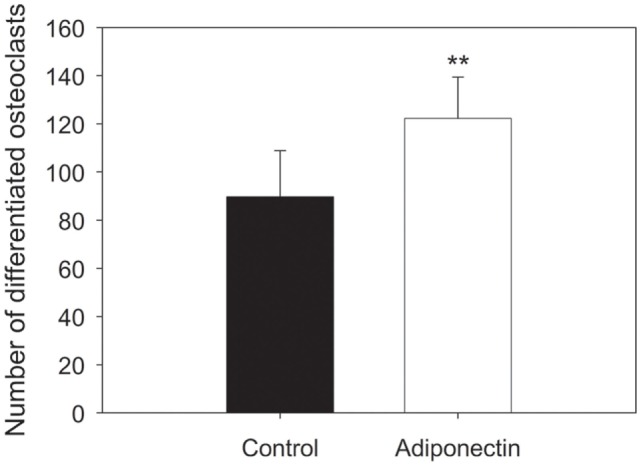
Adiponectin enhanced the number of mature osteoclasts differentiated from human mononuclear cells. Data are presented as mean ± SD (**p < 0.01).

**Figure 11:**
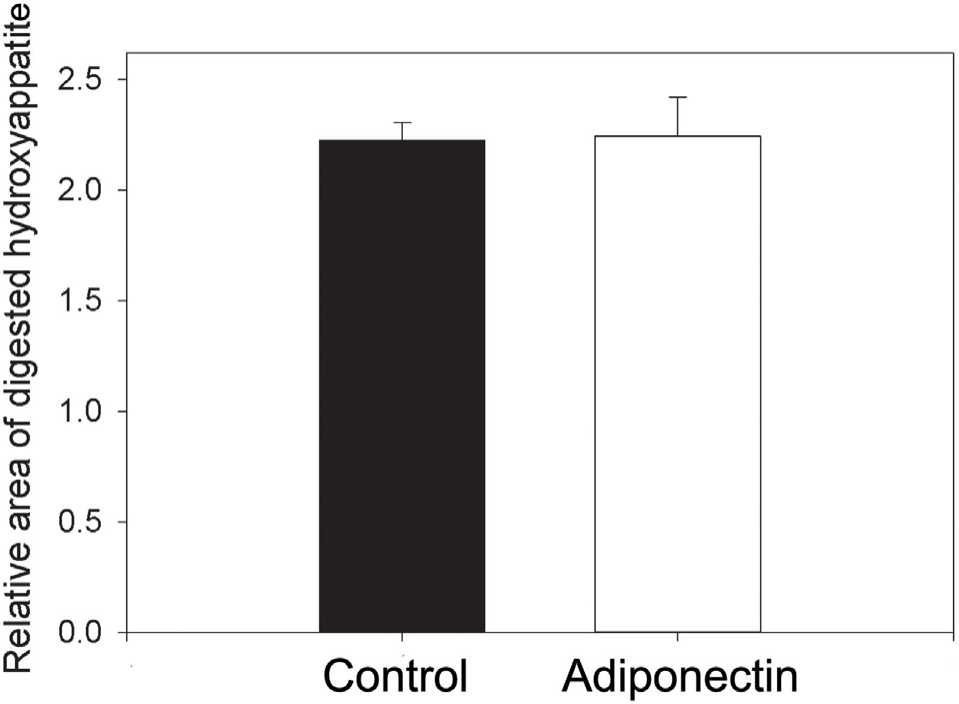
No significant changes were seen in the relative area of digested hydroxyapatite from osteoclasts incubated with and without adiponectin (0.1 µg/mL). Data are presented as mean ± SD.

Evaluation of the number of differentiated osteoclasts after treatment with or without 0.1 µg/mL adiponectin revealed that adiponectin enhanced the differentiation of osteoclasts from human peripheral monocytes compared with untreated cells (*p* < 0.01) (Figure 10). However, the osteoclast activity was not affected by the same dosage of adiponectin compared with control as no significant difference was observed in the relative area of digested hydroxyapatite by osteoclasts incubated with or without adiponectin (Figure 11).

## Discussion

This study demonstrates that adiponectin is associated with reduced bone stiffness, both in osteospheres *in vitro* and in rat femurs *in vivo*. Adiponectin seemed to directly influence bone by regulating proliferation and differentiation of osteoblasts and osteoclasts and alter the expression of ECM proteins.

*In vitro* created tissues are dependent on extracellular scaffolding, and in most models synthetic or non-synthetic biomaterials are used (4, 6, 10, 11). Recently, an interesting model of bone tissue formation *in vitro* has been presented (12). Iordachescu et al. used monocultures of animal bone cells and cell lines, and their model is dependent on a premade fibrin gel cast between scaffolds anchors (12). This is in contrast to the two-cell system presented here, where primary human osteoblasts and osteoclasts are integrated in a spheroid with their secreted ECM proteins. *In vitro* produced osteospheres have the advantage that direct effects on the two primary cell types of bone can be investigated, and in contrast to an *in vivo* setting, complex biological responses may be measured in this simplified model of bone (9).

This method gives the opportunity to investigate effects on mechanical properties. To the best of our knowledge, this is the first report on induced changes in mechanical properties of a human cell-based bone model. Potential limitations in the use of these bone-like tissues may be related to the reproducibility of the shape and size of the constructs. In addition, the distribution of the cells and ECM within the constructs may differ. The osteospheres in this study varied from 0.5 to 1 mm and were significantly smaller, but had the similar morphological features as described by Clarke et al. (9).

We aimed at producing and treating osteospheres with adiponectin at levels within or below physiological range. Plasma levels of adiponectin have been found to be around 6–8 µg/mL in humans (53). Furthermore, Berner et al. found the levels of adiponectin in the bone marrow fluid of mice to be between 0.1 and 0.15 µg/mL. A concentration of 0.01 µg/mL adiponectin was found to enhance the proliferation of human osteoblasts; however, there were no dosage-dependent variations between 0.01 and 2.0 µg/mL of adiponectin on cell growth *in vitro* (30).

Reduced stiffness was observed in adiponectin-treated artificial bone compared with control bone measured by flat punch compression nanoindentation. Conventional nanoindentation with a sharp indenter [pyramidal (Berkovich) or spherical] is a well-established method where the local properties of the bone is investigated to determine the hardness and modulus (54, 55). With this method, the response of the material is measured in a local region below the indenter where the tissue deformations are localized. For soft materials, that kind of indentations can introduce local tissue damage, and consequently it may be difficult to extract the basic material properties describing the stress–strain response for undamaged material. In such cases, the conventional indenter is not applicable, and a flat punch method for compression test has been developed (44, 56). The flat punch method gives the opportunity to avoid local damage from a sharp indenter, and material properties that are more representative for the whole specimen are obtained. Furthermore, the shape of the osteospheres is important with respect to deriving mechanical properties (i.e. stress versus strain). Because of irregular samples, the flat punch method was conducted in this study, and a simplistic analysis was made based on global geometry such as size of equatorial plane and the height of the specimen. In further work, one could establish more detailed finite element models. A disadvantage of the use of spheres in mechanical indentation testing may be the variable inner structure, irregular geometry, and degree of mineralization of the spheres. The relation between adiponectin and reduced stiffness of bone is in accordance with a recent study reporting cortical bone hardness to be 15% higher in 8-week-old APN-KO mice (57).

Adiponectin is abundant in plasma (22) and as previously mentioned epidemiological studies have consistently shown an inverse association between circulating adiponectin and BMD (14, 15), whereas in animal studies the reported effects of adiponectin on bone are inconsistent (13). There are indications that both adipokines and PPAR agonists play important roles in the regulation of bone metabolism (58). Administration of PPAR agonists may have different effects on circulating adiponectin levels in rats. Increased plasma levels of adiponectin in rats given the PPAR gamma agonists pioglitazone (46) and thiazolidinedione (59) have previously been demonstrated, while rats receiving the PPAR alpha agonist fenofibrate exhibited decreased levels (46). Yet, in the protein fractions isolated from flushed femur, the content of adiponectin was similar in the groups receiving different PPAR agonists (46). This study showed an association between the mechanical properties of rat femurs with *in situ* elevated adiponectin expression, supporting that adiponectin is associated with reduced stiffness of bone. In contrast to reports from human studies (14, 15), the effect was not associated with the levels of circulating adiponectin but to the adiponectin mRNA levels locally in femur, indicating an autocrine and/or paracrine effect of adiponectin on bone. A limitation to this study is the low number of samples. Yet, the negative correlation between adiponectin expression and femur stiffness is in accordance with Kajimura and coworkers, who found that bone was more resistant in terms of mechanical strength in APN-KO mice (28).

Adiponectin has previously been reported to enhance osteoblast mineralization (37). We observed no significant difference in mineralization status in 2D osteoblast cultures exposed to low adiponectin dosages compared with unexposed cells, but the same low dosage was shown to reduce stiffness of the osteospheres. The increased proliferation and collagen expression by adiponectin stimulation may indicate that the osteoblasts remain in a proliferative state (60). This is in accordance with a previous study reporting both enhanced osteoblast proliferation and collagen expression after adiponectin stimulation (34). Collagen provides the flexibility (toughness) to the bone structure, contributing to resistance to impact loading (61, 62). The mechanical properties of bone are dependent on both the amount and orientation of collagen fibers (63). Adiponectin has been shown to play a role in wound healing in mice by upregulation of keratin gene transcripts and subsequently promoting collagen organization, thickness, and deposition (64). Also, adiponectin has been shown to enhance the production of collagen III and elastin in fibroblasts (65). The extent to which these findings translate to bone remains to be investigated.

Regarding the effects of adiponectin on osteoclast differentiation and osteoclast activity, diverging results have been reported. Most studies show suppressing effect of adiponectin on osteoclastogenesis (33, 36, 39–41), while others report indirect increase of osteoclast formation *via* RANKL/OPG pathway (42, 43). In this study, recombinant adiponectin enhanced the number of differentiated osteoclasts from primary human monocytes, while no change in activity was observed. Our data are in accordance with Williams et al. who found no effect of adiponectin on osteoclast activity (39), on the other hand, in contrast to Oshima et al. reported suppressing bone-resorption activity of adiponectin-treated osteoclasts (36).

In conclusion, this study indicates that adiponectin is involved in bone stiffness. In addition, we hypothesize that artificial osteospheres produced in an RCCS without scaffolding material may be a model system for testing the effects of different agents on the mechanical features of bone. However, further studies are required.

## Ethics Statement

The experimental protocol was approved by the Norwegian Animal Research Authority (NARA), and the procedures were conducted in accordance with the Animal Welfare Act. The Animal Welfare Committee at St. Olav’s University Hospital in Trondheim approved the study.

## Author Contributions

SH: participated in the experimental design, performed experiments, and drafted the manuscript. AKS, JH, KA, and HT: performed experiments, participated in experimental design, and contributed in editing of the manuscript. AS and US: provided material, participated in experimental design, and contributed in editing of the manuscript. BS: participated in experimental design and contributed in editing of the manuscript. JER: initiated the study, designed the setup of experiments, contributed in drafting and editing of the manuscript.

## Conflict of Interest Statement

The authors declare that the research was conducted in the absence of any commercial or financial relationships that could be construed as a potential conflict of interest.
